# Novel pantothenate derivatives for anti-malarial chemotherapy

**DOI:** 10.1186/s12936-015-0673-8

**Published:** 2015-04-18

**Authors:** Helmi E Pett, Patrick AM Jansen, Pedro HH Hermkens, Peter NM Botman, Christien A Beuckens-Schortinghuis, Richard H Blaauw, Wouter Graumans, Marga van de Vegte-Bolmer, Karin MJ Koolen, Floris PJT Rutjes, Koen J Dechering, Robert W Sauerwein, Joost Schalkwijk

**Affiliations:** Department of Medical Microbiology, Radboud University Nijmegen Medical Center, Nijmegen, The Netherlands; Department of Dermatology and Radboud Institute for Molecular Life Sciences, Radboud University Nijmegen Medical Center, Nijmegen, The Netherlands; Radboud University Nijmegen, Institute for Molecules and Materials, Nijmegen, The Netherlands; Chiralix B V, Nijmegen, The Netherlands; TropIQ Health Sciences, Nijmegen, The Netherlands; Pansynt B V, Nijmegen, The Netherlands

**Keywords:** Malaria, Anti-malarial, Plasmodium falciparum, Pantothenate, Pantothenic acid, Pantothenamide, Pantothenone, Coenzyme A

## Abstract

**Background:**

A number of synthetic pantothenate derivatives, such as pantothenamides, are known to inhibit the growth of the human malaria parasite *Plasmodium falciparum*, by interfering with the parasite Coenzyme A (CoA) biosynthetic pathway. The clinical use of pantothenamides is limited by their sensitivity to breakdown by ubiquitous human pantetheinases of the vanin family.

**Methods:**

A number of pantothenate derivatives (pantothenones) with potent and specific inhibitory activity against mammalian vanins were tested in a proliferation assay of asexual *P. falciparum* blood stages alone, and in combination with pantothenamides.

**Results:**

The vanin inhibitors were found to protect pantothenamides against breakdown by plasma vanins, thereby preserving the *in vitro* anti-malarial activity. Moreover, some of the vanin inhibitors showed *in vitro* anti-malarial activity in the low micromolar range. The most potent antimalarial in this series of compounds (RR8), was found to compete with pantothenate in a combination proliferation assay. No correlation, however, was found between anti-vanin and anti-malarial activity, nor was pantetheinase activity detected in *P. falciparum* extracts.

**Conclusions:**

Growth inhibition is most likely due to competition with pantothenate, rather than pantetheinase inhibition. As vanin inhibitors of the pantothenone class are stable in biological fluids and are non-toxic to mammalian cells, they may represent novel pantothenate-based anti-malarials, either on their own or in combination with pantothenamides.

**Electronic supplementary material:**

The online version of this article (doi:10.1186/s12936-015-0673-8) contains supplementary material, which is available to authorized users.

## Background

Each year over half a million people die of malaria, with *Plasmodium falciparum* being the primary cause of fatal malaria cases [1]. As the eradication of malaria is threatened by occurrence of clinical resistance to artemisinin derivatives, new drugs for malaria are sorely needed and so the search for new lead compounds continues [1].

Based on the observation, that addition of calcium pantothenate to *Plasmodium lophurae* cultures increased parasite viability, a selection of analogues of pantothenate (pantothenic acid, vitamin B5), were tested for antiplasmodial activity as early as the 1940s [2]. These compounds included pantoyltaurine, substituted pantoyltaurylamides, sulphonamides, and pantothenones, according to the nomenclature used in a review on this subject by Spry *et al.* [3]. These and similar compounds were tested in different *in vitro* and *in vivo* malaria models from the 1960s and 1970s [4,5]. In 1976, Trager and Jensen published an article describing the continuous culture of *P. falciparum* [6], allowing Divo *et al.* to discover that pantothenate is indeed the only water soluble vitamin that needs to be exogenously available for *P. falciparum* survival [7]. Meanwhile, Clifton *et al.* prepared a series of analogues with the general structure N1-(substituted) pantothenamide, and found them to have antibacterial activity due to being antimetabolites of pantothenate [8].

Recent studies showed that some of the pantothenamides were also active against *P. falciparum in vitro*, provided that plasma pantetheinase activity was reduced [9]. This was discovered due to the observation that ‘aging’ *P. falciparum* growth media increased the anti-malarial activity of some pantothenamides [9]. Later, this same effect was achieved with heat inactivation of the parasite growth medium by de Villiers *et al.* [10]. The mechanism of breakdown of pantothenamides by pantetheinases of the vanin family was elucidated in detail by Jansen *et al.* who discovered that combining pantothenamides with small molecule vanin inhibitors, protected pantothenamides against breakdown, thereby dramatically increasing their antibacterial activity against both *Staphylococcus aureus* and *Escherichia coli* [11-14]. It has also been shown by de Villiers *et al*. that small modifications of the pantothenamide core structure could protect the molecule against pantetheinase-mediated degradation, albeit at a cost of a 100-fold decrease in anti-malarial potency [10].

Compounds, such as the pantothenamides in *E. coli* or the fungal product CJ-15,801 in *S. aureus* may hijack Coenzyme A (CoA) biosynthesis, being phosphorylated in the first step of the biosynthesis by pantothenate kinase (PanK) and eventually blocking CoA production or interfering with fatty acid synthesis downstream along the pathway [15-17]. Almost a decade ago, the fungal product CJ-15,801, was also discovered to have modest anti-malarial activity against asexual intra-erythrocytic stages of *P. falciparum in vitro*, and was demonstrated to inhibit parasite growth by a mechanism related to CoA biosynthesis or utilization [18].

In this study a selection of novel pantetheine analogues of the pantothenone class were investigated for potential use as anti-malarial chemotherapy. The investigated compounds are shown to be conceptually promising either as a monotherapy or in a combination of drugs.

## Methods

### Compounds

**Figure 1:**
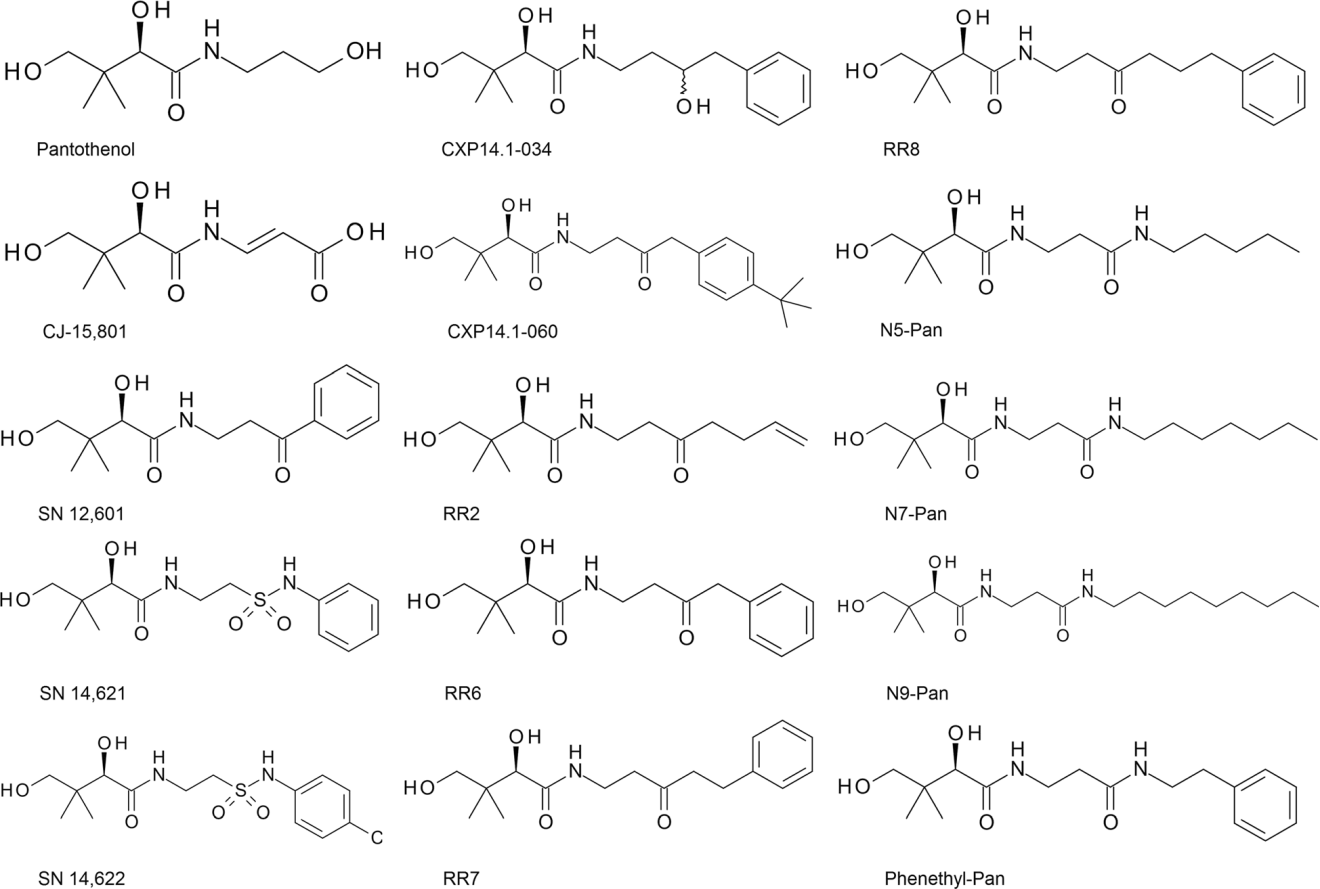
Chemical structures of all the compounds tested in this study.

Pantothenol was purchased from Sigma-Aldrich. Detailed synthesis procedures of CJ-15,801 and CXP14.1-060 are provided as Additional file 1. The methodologies for synthesis of CXP14.1-034, RR2, RR6, RR7, RR8 and SN12,601 have been previously published in Jansen *et al*. [13]. N5-Pan and N7-Pan were synthesized as described in the supplemental material of Jansen *et al.* [14]. N9-Pan was synthesized as N5-Pan and N7-Pan, but instead of using a pentylamine or heptylamine, a nonylamine was used for the synthesis of N9-Pan. The synthesis of SN14,621 and SN14,622 was performed as described in Winterbottom *et al.* [19]. The synthesis of phenethyl-Pan was performed as described by Spry *et al.* [9]. Chemical structures for all of the compounds in this study are presented in Figure 1.

### *Plasmodium falciparum* culture

The asexual stages of the NF54 strain *P. falciparum* were cultured as previously described [6], utilizing a shaker system with automated media change twice a day, parasites were kept in continuous culture within adapted Erlenmeyer flasks [20]. Erythrocytes were refreshed every two or three days to adjust haematocrit to 5% and parasitaemia to 0.5%. Human erythrocytes (blood type A) were obtained from healthy blood donors, with no history of malaria. Culture media consisted of RPMI 1640 with HEPES [5.94 g/l, hypoxanthine [0.05 g/l], 10% (v/v) pooled human serum (blood type A) obtained similarly to erythrocytes, and 0,2% (w/v) sodium bicarbonate. Temperature was set to 37°C and a low oxygen gas mixture was constantly flushed over the culture maintaining a stable atmosphere of 3% O_2_, 4% CO_2_, and 93% N_2_.

### Vanin activity assay

Vanin/pantetheinase activity assay with aminomethyl-coumarine (AMC) substrate and fluorescence readout was performed as described previously in Jansen *et al.* [13,21]. Human serum was used as a source of vanin enzymatic activity in assays to determine the anti-vanin activity of study compounds CJ-15,801, SN 12,601, SN 14,621, SN 14,622, CXP14.1-034, CXP14.1-060, RR2, RR6, RR7, and RR8. To determine whether *P. falciparum* parasites harbour vanin activity, assays were performed on parasite extracts. To this end, 5 to 9 x 10^8^ non-synchronous asexual NF54 strain *P. falciparum* parasites were pelleted by centrifugation at 4000 rpm for 10 minutes. Pellets were re-suspended in 5 ml of 0.06% saponin in phosphate buffered saline (PBS) and incubated on ice for 5 minutes to remove erythrocytes. After this they were washed with PBS twice, with centrifugation in between as when pelleting cultures. Pellets from four different cultures were individually resuspended in a total volume of 300 μl of PBS and lysed by sonication (6 x 3 seconds). Vanin activity was determined by combining 29 μl of lysate with 1 μl of AMC substrate (final concentration of 333 μM) and incubation at room temperature. At 0, 1 and 19 hours, 3 μl of the reaction was diluted with 997 μl of PBS and fluorescence was measured in a 200 μl aliquot. Assay negative controls consisted of 29 μl of PBS and 1 μl of AMC substrate. Positive control consisted of 19 μl of PBS, 10 μl of human serum, and 1 μl of AMC substrate.

### *Plasmodium falciparum* asexual blood stages assay with SYBR Green read-out

A non-synchronous asexual NF54 strain *P. falciparum* culture was adjusted to a parasitaemia of 0.5-1% and a haematocrit of 1-5%. The compound dilution curves were prepared in dimethylsulphoxide (DMSO) from 100 mM to 10 μM at half/log step dilutions. The established anti-malarial dihydroartemisinin (DHA) was used as a positive control and diluted in DMSO from 1 mM to 100 nM at half/log step dilutions. The DMSO dilutions were 500-fold diluted in growth medium to yield a final DMSO concentration of 0.2% (v/v), and final compound concentrations from 200 μM to 20 nM (2 μM to 200 pM for DHA). Higher concentrations for the experimental compounds would not have been possible to achieve due to limitations in final DMSO concentration (0.1% v/v). Fifty μl of each compound dilution was combined with 50 μl of parasite culture in a black, clear-bottomed or entirely black 96-well plate. The outermost wells were filled with sterile water to prevent evaporation. DHA at a concentration of 1 μM and 0.1% DMSO (negative control) were used to determine the assay window. The 96-well plates were incubated for 72 hours in a candle jar at 37°C. Read-out was done using the DNA-marker SYBR Green as described previously [22,23]. All assays were conducted at least in triplicate.

When combining two compounds in the same well the conditions were otherwise similar to the single compound assays, however the final DMSO concentration was 0.2%, which was also adjusted for the positive and negative controls and was not found to cause differences in results obtained with single compounds in assay containing 0.1% DMSO. The pantothenamide phenethyl-Pan was combined with a 10^−7^ to 10^−5^ M concentration curve of CXP14.1-060; the other pantothenamides were combined only with a 5 μM concentration of the same vanin inhibitor. The pantothenone vanin inhibitor RR8 was additionally combined with 20 μM, 6.3 μM, 2.0 μM, and 0.6 μM of pantothenate to explore potential competition for the same molecular target in *P. falciparum*. All assays were conducted at least in triplicate.

### Statistical analyses

Statistical analyses were performed using GraphPad Prism 5. This includes producing inhibition curves, normalizing them to percentage of inhibition, calculation of IC_50_ values, correlation coefficients (Spearman non-parametric correlation), and Schild analyses [24]. The IC_50_ is the concentration of the compound in question at which the compound reaches 50% inhibition relative to the DHA control curve, and is calculated using the interpolate function in GraphPad Prism 5.

## Results

### Anti-malarial activity of pantothenamides

**Figure 2:**
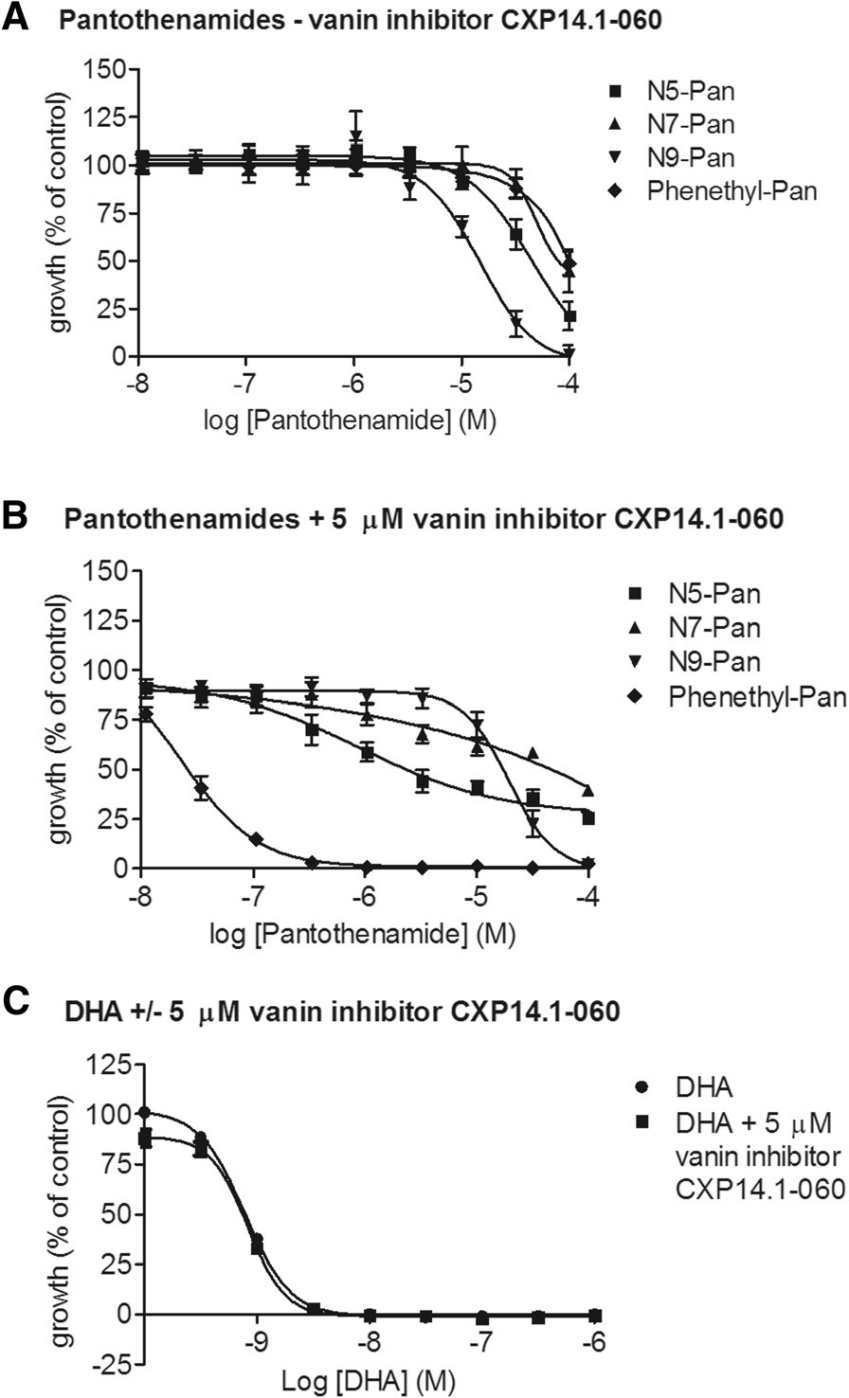
Anti-malarial activity of pantothenamides. Four pantothenamides, phenethyl-Pan, but also N5-Pan, N7-Pan and N9-Pan in asexual blood-stages proliferation assay with SYBR Green read-out, without and in combination with 5 μM of vanin inhibitor CXP14.1-060. **A)** N5-Pan, N7-Pan, N9-Pan and phenethyl-Pan without vanin inhibitor CXP14.1-060. **B)** N5-Pan, N7-Pan, N9-Pan and phenethyl-Pan in combination with vanin inhibitor CXP14.1-060. **C)** DHA with and without 5 μM of vanin inhibitor CXP14.1.-060.

Using *P. falciparum* cultured in 10% human serum, the low level of anti-malarial activity of the pantothenamides N5-Pan, N7-Pan and phenethyl-Pan was confirmed, as shown in Figure 2A. None of these pantothenamides showed an IC_50_ below 50 μM. In addition, N9-Pan was tested. N9-Pan is a pantothenamide that has previously been shown to have modest antibacterial activity but had not been investigated for its anti-malarial activity [8]. N9-Pan was found to be clearly more potent than the other pantothenamides, with an IC_50_ of about 15 μM, in the presence of serum (Figure 2A). To investigate the effect of vanin inhibition on the potency of the pantothenamides N5-Pan, N7-Pan and N9-Pan, these were combined with 5 μM of the newly synthesized vanin inhibitor CXP14.1-060, that has nanomolar potency against human vanins, yet no anti-malarial activity up to the highest concentration tested (100 μM) (Table 1). The potency of N5-Pan and N7-Pan increased upon vanin inhibition, but the effect was not as great as with phenethyl-Pan (Figure 2B). Remarkably, the potency of N9-Pan was not increased by the addition of the vanin inhibitor (Figure 2B). The potency of DHA did not change upon addition of 5 μM of the vanin inhibitor CXP14.1-060 (Figure 2C).

**Table 1:** **IC**_**50**_ **values for individual compounds**

	**Anti-malarial activity**		Anti-vanin activity*
**Compound**	**IC**_**50**_ **(μM)**	**95% C.I. (μM)**	**IC**_**50**_ **(μM)**
Pantothenol	>100	NA	NT
CJ-15,801	>100	NA	213.60
SN 12,601	6.1	4.5-8.7	17.61
SN 14,621	33.6	7.3-NA	33.70
SN 14,622	7.4	3.5-20.7	31.89
CXP14.1-034	24.1	11.3-NA	1.69
CXP14.1-060	>100	NA	0.039
RR2	2.6	2.1-3.3	2.85
RR6	14.5	5.3-NA	0.04
RR7	7.7	5.1-12.6	0.11
RR8	2.2	1.6-3.1	0.27
N5-Pan	45.1	34.8-60.2	NT
N7-Pan	79.0	34.0-NA	NT
N9-Pan	14.6	11.0-20.1	NT
Phenethyl-Pan	98.2	NA	NT

*Anti-vanin activity tested in human serum.

NA: Not Applicable.

NT: Not Tested.

**Figure 3:**
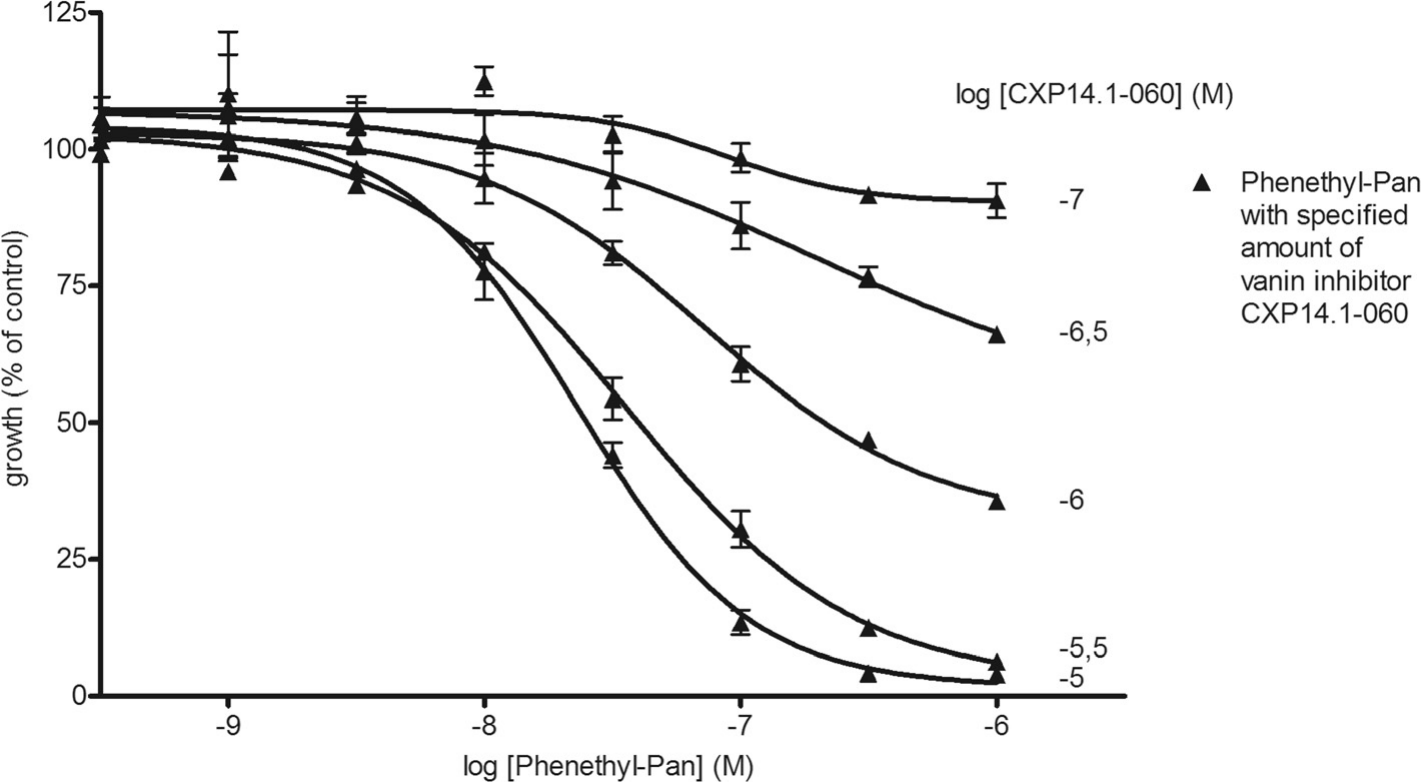
Anti-malarial activity of phenetyl-Pan in the presence of varying concentrations of vanin inhibitor CXP14.1-060. Combination of anti-malarial pantothenamide phenethyl-Pan [9] with novel vanin inhibitor CXP14.1-060. Filled upright triangles represent phenethyl-Pan, with CXP14.1-060 added at a different concentration for every curve as indicated in figure. At a concentration of 10 μM of CXP14.1-060 phenetyl-Pan has an IC_50_ of 23 nM (95% CI: 16.5-31.9 nM).

Phenethyl-Pan was selected to further investigate the protective effect of the synthetic vanin inhibitor CXP14.1-060 on its anti-malarial activity. Phenethyl-Pan was previously shown to have an IC_50_ of 20 nM in ‘aged’ medium and is the most potent anti-malarial pantothenamide known so far [9]. A dose range of this compound was tested in the presence of varying concentrations of CXP14.1-060 (Figure 3). The potency of phenethyl-Pan visibly improved upon addition of increasing concentrations of CXP14.1-060, reaching an IC_50_ of 23 nM (95% CI: 16.5-31.9 nM) upon addition of 10 μM of CXP14.1-060 (Figure 3). In the presence of 10 μM of vanin inhibitor CXP14.1-060, phenethyl-Pan showed a full inhibition of parasite growth, comparable to the efficacy of DHA, which was used as a reference compound and has an IC_50_ in the low nanomolar range. This experiment shows that small molecule inhibition of pantothenamide hydrolysis will allow the same level of protection found in heat-inactivated medium as shown by the comparable IC_50_ values [10].

### Anti-malarial activity of pantothenones

**Figure 4:**
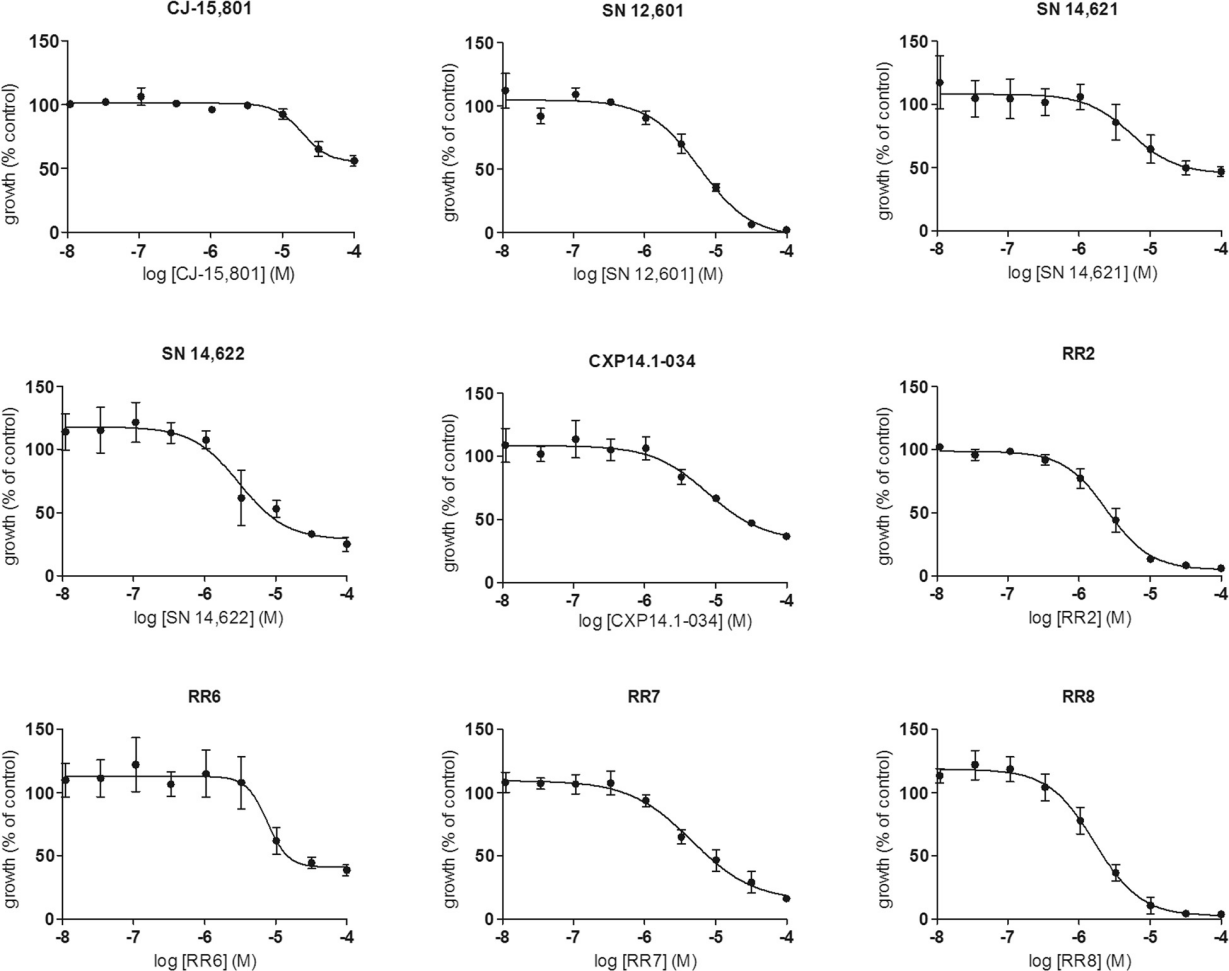
Anti-malarial activity of pantothenones and reference compounds. Inhibition curves for all compounds with IC_50_ values below 100 μM, and CJ-15,801, against asexual blood stages of *P. falciparum* in a proliferation assay with a SYBR Green read-out.

15 pantothenate derivatives were synthesized and assayed for anti-malarial activity in the in the presence of 10% human serum. The structures of these compounds are presented in Figure 1, and their observed individual biological effects are presented in Table 1. Some of the newly synthesized vanin inhibitors were structurally similar to pantothenate derivatives described in the 1940s, which were shown to have anti-malarial activity in avian malaria models [2]. Some of these reference compounds were re-synthesized, including the pantothenone SN12,601 and the sulphonamides SN 14,621 and SN 14,622. In this study, they were found to have moderate activity, and the effect of their dilution curves on *P. falciparum* growth are shown in Figure 4. In addition, pantothenol and CJ-15,801, two natural compounds with known weak activity against *P. falciparum*, were also tested [18,25]. CJ-15,801 was found to be a poor inhibitor of *P. falciparum* growth (Table 1 and Figure 4) and pantothenol showed no anti-malarial activity up to the highest concentration tested (100 μM) (Table 1). Out of the newly synthesized pantothenones, the vanin inhibitor RR8 was the most potent of these compounds, having an IC_50_ value of 2.2 μM (95% CI: 1.6-3.1 µM) (Table 1 and Figure 4).

### Mechanism of anti-malarial activity of pantothenones

**Figure 5:**
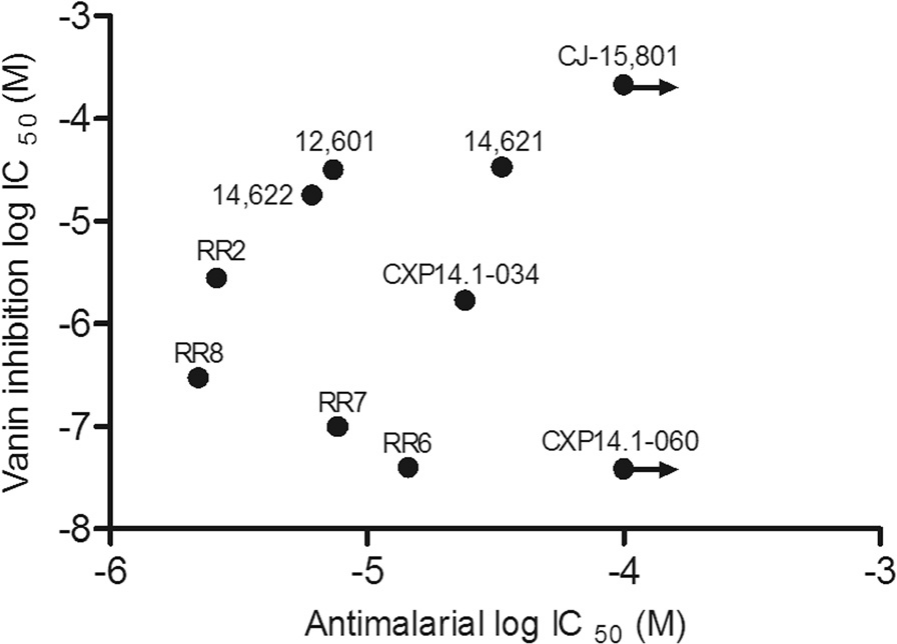
Lack of correlation between anti-malarial activity and anti-vanin activity. The IC_50_ for inhibition of vanin activity in human serum was plotted against the anti-malarial IC_50_ in asexual blood stages proliferation assay of *P. falciparum* with SYBR Green read-out. No significant correlation was observed. (Spearman rho=0.06079 (p=0.8651)).

**Figure 6:**
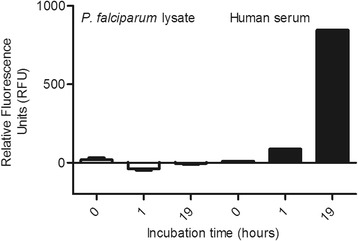
Lack of vanin activity in *P. falciparum* lysates. Vanin activity assays were performed using a fluorescent aminomethylcoumarine (AMC) substrate on lysates of *P. falciparum* cultures (on the right in white), and as a positive control human serum (on the left in black). The figure shows mean relative fluorescence units and standard deviations from measurements of four independent *P. falciparum* cultures. Values were corrected for background fluorescence signals measured in negative control (PBS) samples.

**Figure 7:**
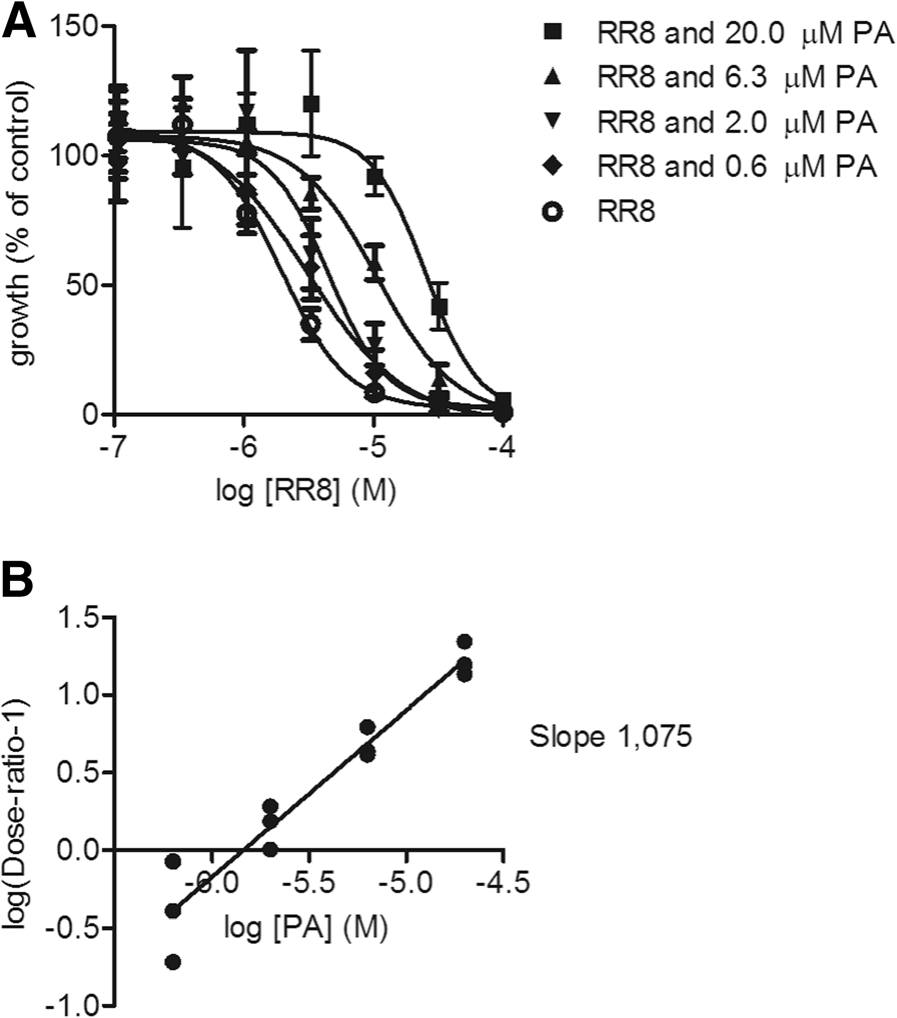
Pantothenate competes with RR8. Competition assay between RR8 and pantothenate (PA), results of Schild analysis. **A)** Shifting IC_50_: Combined data points from three experiments. RR8 with 20.0 μM PA, RR8 with 6.3 μM PA, RR8 with 2.0 μM PA, RR8 with 0.6 μM PA and RR8 alone. **B)** Schild plot: Combined data points from three experiments. A slope of 1 is indicative of a competitive antagonistic relationship between RR8 and PA.

It was considered, that the pantetheinase inhibiting activity of the new compounds may contribute to anti-malarial activity. Therefore, the IC_50_ values of *in vitro* inhibition of asexual blood stages of *P. falciparum* were plotted against the IC_50_ values of anti-vanin activity in human serum (Figure 5). There was no correlation between these activities, suggesting that pantetheinase inhibition is not the mode of action of the small molecule inhibitors in *P. falciparum* asexual blood stages. In line with this finding, an assay using the AMC substrate to detect pantetheinase activity, did not detect hydrolytic activity in extracts of purified asexual stages of *P. falciparum*, even after a 19 h incubation period, suggesting that the parasite lacks such enzyme activity (Figure 6). This notion was corroborated by a BLAST search that did not reveal sequences in the *P. falciparum* genome database with homology to mammalian vanin genes. In order to investigate an alternate mode of action, competition experiments with pantothenate were performed. To this end, a *P. falciparum* asexual blood stage growth assay was performed with eight concentrations of RR8 combined with eight concentrations of pantothenate. An increase in the IC_50_ of RR8 was found to occur at addition of increasing concentrations of pantothenate (Figure 7A). Upon performing a Schild analysis on the results of the concentrations, 20 μM, 6.3 μM, 2.0 μM, and 0.6 μM of pantothenate in combination with a dilution curve of RR8, the slope of the line in the Schild Plot was 1.087 ± 0.089, indicating that RR8 and pantothenate are competing for the same target in *P. falciparum* (Figure 7B).

## Discussion

This study underscores the potential of pantothenate derivatives for anti-malarial therapy, and demonstrates that the most potent serum-labile anti-malarial pantothenamide (phenethyl-Pan) can be effectively protected against hydrolysis by serum pantetheinases using the novel vanin inhibitor CXP14.1-060. From a mechanistic point of view, this study indicates that the pantothenone RR8 exerts its anti-malarial effect through competition with pantothenate.

In the 1940s, a number of chemical variations on pantothenate were synthesized and tested for anti-malarial activity [2]. These included pantothenones and sulphonamides, which were found to be active against avian malaria. This study shows that these compounds are also active against the human parasite *P. falciparum*.

Although the new vanin inhibitor CXP14.1-060 effectively protected phenethyl-Pan, such a combination of drugs would be undesirable from a drug development perspective. Clearly, the potency and/or stability of pantothenate derivatives needs to be improved before they can enter a drug development programme as therapeutic agents for human malaria infection. Nevertheless, the recent discovery of phenethyl-Pan with an IC_50_ of 20 nM is encouraging [9]. Although this compound is unstable in plasma, it illustrates that it is realistic and feasible to aim for pantothenate derivatives active in the low nanomolar range. The study by de Villiers *et al.* showed that structural modifications of pantothenamides can be introduced to confer resistance to plasma-mediated breakdown [10]. These novel compounds, although less potent than the original pantothenamides, are a starting point for further lead optimization studies [10]. Optimization of the potency would be important to maximize the risk-benefit of a novel drug, as side effects may be mediated by low-affinity, off-target effects. In addition, increasing the potency may lead to a lower effective dose in humans, and hence impact the cost of treatment. Availability of affordable medicines is an important driver for success in malaria control, and the goal for development of novel drug therapies is to achieve effective treatment with a total cost of US$1 [26]. In that respect, the molecules described here provide attractive candidates as their chemistry is simple, which ensures a low cost of goods in a manufacturing process.

Many of the marketed anti-malarials and compounds in the clinical development portfolio originate from whole cell phenotypic screening efforts and exert their actions by inhibiting multiple targets or pathways of the parasite. Although such a polypharmacological profile may be important to their efficacy, it is an undesirable feature in a rational medicinal chemistry approach. The exact target of the anti-malarial pantothenate derivatives has not been identified unequivocally but is it likely that they exert their effects by affecting targets dependent on pantothenate. In theory, the observed effects could still be mediated by effects on red blood cell biology (e.g., red blood cell pantothenate kinases (PANK)) rather than directly on the parasite. However, the recent discovery of a parasite-specific pantothenate transporter [27] leaves very little doubt that the parasite itself is the target of interfering with pantothenate dependent pathways. Future drug development efforts would benefit from information on the molecular targets of pantothenamides, which would include both biosynthetic pathways (CoA synthesis, lipid synthesis, energy metabolism) and pantothenate transport systems. Structural information, which is available for mammalian and bacterial PANK, could guide medicinal chemistry strategies to achieve specific inhibition of the parasite enzyme and reduce side effects on the host.

## Conclusions

Pantothenamides with anti-malarial activity can be protected from breakdown by ubiquitous pantetheinases of the vanin family with small molecule pantothenone vanin inhibitors. Some of these pantothenones exhibit anti-malarial activity in their own right. Compound series such as the one tested in this publication should be studied further for use as lead compounds for anti-malarial treatment.

## Additional file

Additional file 1:
**Supplemental Materials and Methods.** The supplemental materials and methods explain how the compounds CJ-15,801 and CXP14.1-060 were synthesized.

## Abbreviations

CoA: Coenzyme A
DHA: Dihydroartemisinin
DMSO: Dimethylsulphoxide
PA: Pantothenic acid, pantothenate, vitamin B5
PanK: Pantothenate kinase (*Plasmodium falciparum*)
PANK: Pantothenate kinase (*Homo sapiens*)

## References

[1] WHO (2013). World malaria report 2013.

[2] Wiselogle FY (1946). A survey of antimalarial drugs.

[3] Spry C, Kirk K, Saliba KJ (2008). Coenzyme a biosynthesis: an antimicrobial drug target. FEMS Microbiol Rev.

[4] Trager W (1966). Coenzyme a and the antimalarial action *in vitro* of antipantothenate against *plasmodium lophurae*, *P. Coatneyi* and *P. Falciparum*. Trans N Y Acad Sci.

[5] Trager W (1971). Further studies on the effects of antipantothenates on malaria parasites (*Plasmodium coatneyi* and *P. falciparum*) *in vitro*. J Protozool.

[6] Trager W, Jensen JB (1976). Human malaria parasites in continuous culture. Science.

[7] Divo AA, Geary TG, Davis NL, Jensen JB (1985). Nutritional requirements of *Plasmodium falciparum* in culture. I. Exogenously supplied dialyzable components necessary for continuous growth. J Protozool.

[8] Clifton G, Bryant SR, Skinner CG (1970). N'-(substituted) pantothenamides, antimetabolites of pantothenic acid. Arch Biochem Biophys.

[9] Spry C, Macuamule C, Lin Z, Virga KG, Lee RE, Strauss E (2013). Pantothenamides are potent, on-target inhibitors of *Plasmodium falciparum* growth when serum pantetheinase is inactivated. PLoS One.

[10] de Villiers M (2013). Structural modification of pantothenamides counteracts degradation by pantetheinase and improves antiplasmodial activity. ACS Med Chem Lett.

[11] Jansen PA, Zeeuwen PL, Schalkwijk J, Rutjes FP, Ritzen B, Hermkens PH. Pantothenic acid derivatives and their use in the treatment of microbial infections. Patent Application number EP11725211, Publication number WO2011152720. 2011.

[12] Jansen PA, Schalkwijk J, Rutjes FP, Sauerwein R, Hermkens PH. Derivatives of pantothenic acid and their use for the treatment of malaria. Patent application number EP11725211, publication number WO2011152721. 2011.

[13] Jansen PA, van Diepen JA, Ritzen B, Zeeuwen PL, Cacciatore I, Cornacchia C (2013). Discovery of small molecule vanin inhibitors: new tools to study metabolism and disease. ACS Chem Biol.

[14] Jansen PA, Hermkens PH, Zeeuwen PL, Botman PN, Blaauw RH, Burghout P (2013). Combination of pantothenamides with vanin inhibitors as a novel antibiotic strategy against Gram-positive bacteria. Antimicrob Agents Chemother.

[15] Strauss E, Begley TP (2002). The antibiotic activity of N-pentylpantothenamide results from its conversion to ethyldethia-coenzyme a, a coenzyme a antimetabolite. J Biol Chem.

[16] Zhang YM, Frank MW, Virga KG, Lee RE, Rock CO, Jackowski S (2004). Acyl carrier protein is a cellular target for the antibacterial action of the pantothenamide class of pantothenate antimetabolites. J Biol Chem.

[17] van der Westhuyzen R, Hammons JC, Meier JL, Dahesh S, Moolman WJ, Pelly SC (2012). The antibiotic CJ-15,801 is an antimetabolite that hijacks and then inhibits CoA biosynthesis. Chem Biol.

[18] Saliba KJ, Kirk K (2005). CJ-15,801, a fungal natural product, inhibits the intraerythrocytic stage of *Plasmodium falciparum in vitro* via an effect on pantothenic acid utilisation. Mol Biochem Parasitol.

[19] Winterbottom R, Clapp JW, Miller WH, English JP, Roblin RO (1947). Studies in chemotherapy; amides of pantoyltaurine. J Am Chem Soc.

[20] Ponnudurai T, Lensen AH, Meis JF, Meuwissen JH (1986). Synchronization of *Plasmodium falciparum* gametocytes using an automated suspension culture system. Parasitology.

[21] Ruan BH, Cole DC, Wu P, Quazi A, Page K, Wright JF (2010). A fluorescent assay suitable for inhibitor screening and vanin tissue quantification. Anal Biochem.

[22] Smilkstein M, Sriwilaijaroen N, Kelly JX, Wilairat P, Riscoe M (2004). Simple and inexpensive fluorescence-based technique for high-throughput antimalarial drug screening. Antimicrob Agents Chemother.

[23] Bennett TN, Paguio M, Gligorijevic B, Seudieu C, Kosar AD, Davidson E (2004). Novel, rapid, and inexpensive cell-based quantification of antimalarial drug efficacy. Antimicrob Agents Chemother.

[24] Wyllie DJ, Chen PE (2007). Taking the time to study competitive antagonism. Br J Pharmacol.

[25] Saliba KJ, Ferru I, Kirk K (2005). Provitamin B5 (pantothenol) inhibits growth of the intraerythrocytic malaria parasite. Antimicrob Agents Chemother.

[26] Burrows JN, van Huijsduijnen RH, Mohrle JJ, Oeuvray C, Wells TN (2013). Designing the next generation of medicines for malaria control and eradication. Malar J.

[27] Augagneur Y, Jaubert L, Schiavoni M, Pachikara N, Garg A, Usmani-Brown S (2013). Identification and functional analysis of the primary pantothenate transporter, PfPAT, of the human malaria parasite *Plasmodium falciparum*. J Biol Chem.

