# Supplementary material for: Novel pantothenate derivatives for anti-malarial chemotherapy
Source: Malar J. 2015 Apr 18;14:169. doi: 10.1186/s12936-015-0673-8 (PMC4425855; doi:10.1186/s12936-015-0673-8)
Supplement: Additional file 1: — Supplemental Materials and Methods. The supplemental materials and methods explain how the compounds CJ-15,801 and CXP14.1-060 were synthesized. [file 12936_2015_673_MOESM1_ESM.pdf]

Supplemental materials and methods

General remarks

Standard syringe techniques were applied for the transfer of air sensitive reagents and dry solvents. Reactions were followed, and R_f values are obtained using thin layer chromatography (TLC) on silica gel-coated plates (Merck 60 F254) with the indicated eluent and compounds were detected with UV-light and/or by charring at ca. 150 °C after dipping into a solution of potassium permanganate. Column chromatography was carried out using ACROS silica gel (0.035-0.070 mm, pore diameter ca. 6 mm). ^{1}H NMR spectra were recorded at 298 K on a Varian 400 (400 MHz) spectrometer in $CDCl_3$ or CD_3OD. Chemical shifts are given in parts per million (ppm) with respect to tetramethylsilane (0.00 ppm), as internal standard for $CDCl_3$, or with respect to the solvent residual signal; 3.11 ppm for CD_3OD. Coupling constants are reported as J values in hertz (Hz).

Synthetic scheme for compound CJ-15,801

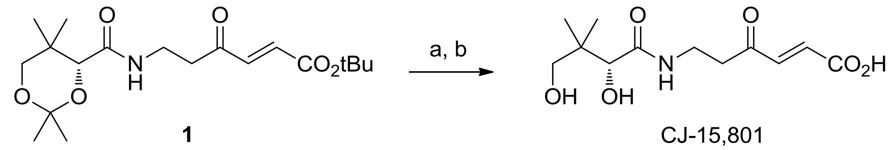

1 CJ-15,801

Reagents and conditions: (a) PTSA, methanol/H_2O (2:1), rt, 93%; (b) *i*: formic acid, *ii*: K_2CO_3, MeOH, rt. 50%

CJ-15,801:

A solution of **1** (400 mg, 1.27 mmol)[1] in a mixture of MeOH/water (ratio 2:1, 12 mL) was treated with PTSA (19.4 mg, 0.10 mmol). After 18 h, more PTSA (15.0 mg, 0.09 mmol) was added. The mixture was stirred of another 4 h before it was diluted with water (80 mL) and EtOAc (80 mL). The organic phase was separated and washed with saturated aqueous $NaHCO_3$ (80 mL), brine (80 mL) and dried over Na_2SO_4. After evaporation of the solvents the product was obtained as a white solid (325 mg, 93%). R_f = 0.32 (EtOAc/heptane = 3:1).

Step b: The product of step a (225 mg, 0.82 mmol) was dissolved in formic acid (3 mL) and stirred for 2 h. The solvent was evaporated and once co-evaporated with toluene. The remainder was dissolved in MeOH (5 mL) and K_2CO_3 (124 mg, 0.90 mmol) was added. After 1 h, the mixture was diluted with water (30 mL) and EtOAc (40 mL). The mixture was acidified to pH 3 by addition of 0.1 M aqueous HCl (~10 mL). The aqueous layer was extracted with EtOAc (2 × 50 mL). The organic phases were combined and washed with brine (50 mL), dried over Na_2SO_4 and filtered. After the solvent was evaporated the product was purified by flash column chromatography (EtOAc/heptane = 2:1 → 8:1, all with 0.5% AcOH). The product was obtained as a colourless oil (88 mg, 50%). R_f = 0.17 (EtOAc/heptane = 3:1); ^{1}H NMR (CD_3OD): δ 7.93 (d, 1H, J = 14.2 Hz); 5.69 (d, 1H, J = 14.2 Hz); 4.01 (s, 1H); 3.49 (d, 1H, J = 10.8 Hz); 3.38 (d, 1H, J = 10.8 Hz); 0.94 (s, 3H); 0.93 (s, 3H). ^{1}H NMR complies with the reported spectrum.[1]

Synthetic scheme for compound CXP14.1-060

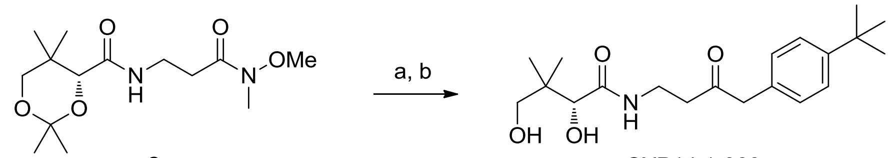

2 CXP14.1-060

Reagents and conditions: (a) (4-(*tert*-butyl)benzyl)magnesium bromide, Et_2O, 0 ºC to rt; (b) TsOH, MeOH, H_2O, rt.

CXP14.1-060:

Step a: Magnesium (221 mg, 9.10 mmol) was stirred in dry Et_2O (20 mL) and 1-(bromomethyl)-4-(tert-butyl)benzene (1.56 mL, 8.27 mmol) was added, followed by 1,2-dibromoethane (0.03 mL, 0.33 mmol) and a catalytic amount of iodine. The hazy solution was stirred at reflux temperature for 45 min. The Grignard reagent (5.61 mL, 3.31 mmol) added dropwise to a cooled (0 °C) solution of Weinreb amide **2** (250 mg, 0.82 mmol)[2] in Et_2O (1.5 mL) over a period of 10 min. The mixture was allowed to warm up to room temperature after 15 min. The reaction mixture was stirred for 2 h and quenched with saturated aqueous NH_4Cl (35 mL) and diluted with EtOAc (80 mL). The organic phase was washed with saturated aqueous NH_4Cl (20 mL), H_2O (20 mL) and brine (20 mL), dried over Na_2SO_4, filtered and the solvent was evaporated. After purification by flash column chromatography (EtOAc/heptane = 1:2 → 2:1) the product was obtained as a colourless oil (47 mg, 15%). R_f = 0.52 (EtOAc/heptane = 3:1); ^{1}H NMR ($CDCl_3$): δ 7.36-7.32 (m, 2H), 7.13-7.10 (m, 2H), 6.90 (br s, 1H), 4.02 (s, 1H), 3.66 (d, 1H, *J* = 11.7 Hz), 3.65 (s, 2H), 3.56-3.38 (m, 2H), 3.25 (d, 1H, *J* = 11.7 Hz), 2.78-2.64 (m, 2H), 1.45 (s, 3H), 1.40 (s, 3H), 1.31 (s, 9H), 1.00 (s, 3H), 0.87 (s, 3H).

Step b: All material of step a (47 mg, 0.12 mmol) was dissolved in MeOH (0.75 mL) and H_2O (0.25 mL) and TsOH·H_2O (1.8 mg, 9.6 µmol) were added. The mixture was stirred for 16 h at room temperature and the same amount of TsOH·H_2O was added. After 4 h, the mixture was diluted with H_2O (10 mL) and EtOAc (15 mL). The organic layer was washed with saturated aqueous $NaHCO_3$ (10 mL) and brine (10 mL), dried over Na_2SO_4 and concentrated *in vacuo*. The product was co-evaporated twice with DCM, yielding CXP14.1-060 as a colorless oil (41 mg, 98%). R_f = 0.21 (EtOAc/heptane = 3:1); ^{1}H NMR ($CDCl_3$): δ 7.37-7.33 (m, 2H), 7.14-7.11 (m, 2H), 7.03 (br s, 1H), 3.92 (d, 1H, *J* = 4.8 Hz), 3.66 (s, 2H), 3.51 (d, 1H, *J* = 11.7 Hz), 3.50 (d, 1H, *J* = 11.9 Hz), 3.46-3.36 (m, 2H), 3.25 (d, 1H, *J* = 5.1 Hz), 2.86 (br s, 1H), 2.78-2.69 (m, 2H), 1.31 (s, 9H), 0.96 (s, 3H), 0.82 (s, 3H).

References

1. Sewell, A.L. et al. *Org. Lett.* **13**, 800-803 (2011).
2. Jansen, P.A.M. et al. *ACS Chem. Biol.* **8**, *530−534* (2012).
